# A national exploration of the utilisation of nurse prescribing in Finland: a cross-sectional survey in outpatient settings

**DOI:** 10.1186/s12912-026-04354-z

**Published:** 2026-02-07

**Authors:** Susanna Nylund, Auvo Rauhala, Erika Boman, Heli Vaartio-Rajalin, Lisbeth Fagerström

**Affiliations:** 1 Department of Natural and Health Sciences, The Faculty of Science and Engineering, Åbo Akademi University, Rantakatu 2, Vaasa, FI-65100 Finland; 2 Department of Research and Development, Åland University of Applied Sciences, Högskolan på Åland, PB 1010, Mariehamn Åland Islands, AX-22111 Finland; 3 Master School/Health and Wellbeing, Turku University of Applied Sciences, Joukahaisenkatu 3, Turku, 20520 Finland

**Keywords:** Clinical competence, Cross-sectional studies, Prescriptions, Finland, Nurses, Outpatient care, Practice patterns, nurses´, Professional autonomy, Surveys and questionnaires

## Abstract

Nurse prescribing is an advanced competency that gives nurses the authority to prescribe medication. Nurse prescribing is increasing globally in response to increasing healthcare demands. There is limited research on nurse prescribing in Finland and other European countries that apply limited nurse prescribing models. This study aimed to explore factors associated with the utilisation of nurse prescribing in Finland. This cross-sectional survey included 84 Finnish registered nurse prescribers. Participants were invited by email and trough snowball sampling. Data were collected via an online questionnaire and analysed using descriptive statistics and multiple linear regression. The study followed the guidelines of Strengthening the Reporting of Observational Studies in Epidemiology for cross-sectional studies. Prescribing consultations of longer durations were significantly associated with a higher degree of utilisation of nurse prescribing. There was also an association between prescribing consultation duration and the work experience of prescribing nurses, with those who had been prescribing longer than 3.4 years holding shorter consultations and having a higher weekly number of patients who were prescribed medications. A background education longer than the registered nurse education was associated with higher accuracy of patient referrals to nurse prescriber clinics and a higher frequency of patients who were prescribed medication. This study contributes to the understanding of the factors affecting the extent of the utilisation of nurse prescribing, including prescribing patterns, in countries that follow a limited nurse prescribing model. The registered nurse prescribers reported a moderately high degree of nurse prescriptive utilisation, although the volume of weekly patients who were prescribed medication was low. In the early stages of their prescribing careers, registered nurse prescribers should be provided with adequate support to increase the level of utilisation of nurse prescribing. The results have implications for how to increase the low utilisation of nurse prescribing among early-career registered nurse prescribers.

## Background

Nurse prescribing enables an expanded nursing role [1]. It is a positive response to global healthcare challenges driven by the necessity for the evolution of healthcare services and the roles of health professionals, including a greater need for access to healthcare resources for a rapidly growing older population with a longer life expectancy [2].

The introduction of nurse prescribing in the United States in the 1970s [1] was an important and pioneering initiative in the modern history of nursing [3]. By 2021, nurse prescribing had expanded to at least 44 countries, 14 of which were in Europe [1]. Today, a wide range of nurse prescribing models with various levels of responsibility exist to regulate the nurse prescriptive authority [1]—that is, the legal extent of prescribing. Generally, prescribing models have evolved according to factors and health needs in local contexts. Consequently, there are no uniform international regulations for nurse prescribing [4]. Nurse prescribing models range from independent prescribing, with a high level of responsibility concerning clinical decisions and treatment, to more limited models that restrict the level of responsibility in various ways [1]. The length of education required before a nurse can prescribe medication varies from the diploma level to a master’s or doctoral degree in advanced practice nursing [1]. In the European context, fully independent nurse prescribing models have been implemented only in Ireland, the Netherlands and the United Kingdom, while other countries, including Finland, have applied only limited prescribing models [5].

There is limited research evidence on nurse prescribing in the European context [5], especially in the case of Finland and other European countries such as Cyprus, Denmark, Estonia, France, Iceland, Norway, Poland, Spain, Sweden and Switzerland, which follow a limited nurse prescribing model. Less than half of these 11 countries have published research on nurse prescribing internationally since 2012 [6–13].

The few primary studies on prescribing patterns in European countries that follow limited prescribing models were conducted in Finland, Norway, Spain and Sweden [7–10]. Experiences in European countries that follow limited prescribing models demonstrate that nurse prescribing increases patients’ access to prescriptions [10, 11]. However, the level of utilisation of nurse prescribing, the amount of prescribed medication [8–10, 12] and the extent of the prescribing models vary among countries [5]. Norwegian public health nurses and midwives with prescriptive authority prescribe more contraceptives than physicians do for women aged 17 and 18 years old [10]. In contrast, the utilisation of nurse prescribing among Swedish public health nurses is generally low, with few medications being prescribed [8]. Studies from Spain show that prescribing efficiency can be affected by nurse experience, with early-career Spanish prescribing nurses prescribing fewer medications [9, 12].

Furthermore, less effective nurse prescribing has been associated with low self-efficacy [12], insufficient nurse prescribing education, lack of organisational support and a restrictive prescribing model with respect to highly restricted prescribing options and dependence on medical professionals [13]. Conversely, higher organisational empowerment, more opportunities to stay updated with prescribing practices and support from leaders and colleagues are related to higher prescribing frequency [8].

There is a need for more research on the effects of Finnish nurse prescribing services from different perspectives [6, 14], including the efficiency of nurse prescribing on outpatient healthcare services and organisations [6, 15] and how to better utilise nurse prescribers’ competence [7]. The rationale for our study is that Finnish nurse prescribers lack opportunities to utilise their nurse prescriptive authority to its full potential due to the limitations of the prescribing model [16] and the increasing number of remote consultations in the healthcare sector [17]. Patients who benefit from nurse prescribing services should be referred to nurse prescribers so that the nurse prescribers advanced competence can be fully utilised.

## Finnish nursing education and roles

The higher education system in Finland includes universities and universities of applied sciences (UAS) [18]. The nursing education provided at UAS consists of four bachelor’s degrees in nursing: nursing (210 European Credit Transfer and Accumulation System [ECTS]), public health nursing, emergency care nursing and midwifery (ranging from 240 to 270 ECTS) [19]. The postgraduate degrees for nurses are UAS or university master’s degrees, with the possibility of continuing to a doctoral degree at a university [20]. Another pathway for nurses in working life is specialisation studies (30–60 ECTS) [20].

Nurses with a bachelor’s degree provide and develop evidence-based care, health promotion and rehabilitation for patients in different healthcare contexts [20]. These also include midwives and public health nurses who practice independently at an advanced level [18]. Nurses with a specialisation also provide independent clinical consultations for patients within their own specialist areas. In Finland three advanced clinical roles have been identified at the master’s level: clinical nurse specialist, nurse practitioner [20], and clinical nursing science specialist [21].

Finnish postgraduate nurse prescribing education is jointly arranged by UAS and universities, comprises 45 ECTS and follows national legislation [22]. To access nurse prescribing education in Finland, the applicants must be experienced licensed registered nurses (RN) [22] or public health nurses, midwives and paramedics with a RN licensing and registration [23]. Supervision and assessment by a physician are required for nurse prescribing students during teaching in practice [22].

## The Finnish limited nurse prescribing model

Finland introduced nurse prescribing in 2010, owing to a reform of primary healthcare, which included a task shift from physicians to other health professionals [24]. Nonmedical professionals with prescriptive authority also include dental hygienists and opticians who prescribe within their scope of practice [16]. Only physicians and dentists have the authority to diagnose and make medical decisions regarding examinations and treatments [23].

In this study, the Finnish nurse prescribing model is described according to legislation at the time of data collection in 2021. The limited model includes a list of medications and indications that can be prescribed to patients who fulfil the criteria specified in the national regulation [16]. Nurse prescribing is allowed in public outpatient services [25]. According to legislation [23], nurse prescribers have the authority to start treatment based on their own assessment of the patient’s symptoms and needs. They can prescribe according to an order from the physician in charge of the organisation in which they are employed. Nurse prescriptive authority entails independently prescribing according to the order for preventive care or symptomatic treatment needs [16]. It also gives nurses the authority to continue prescribing medication for long-term health needs when a physician has diagnosed a patient, prescribed medication and formulated a treatment plan [19]. To prescribe, a nurse prescriber must meet a patient face-to-face and assess the need for treatment, except when initiating treatment with antibiotics for a urinary tract infection, which can be prescribed based on a telephone consultation [26]. There is no official title for Finnish nurse prescribers [19]; however, the concept of ‘nurses with limited prescription rights’ is commonly used to describe advanced competency. This study uses the term ‘registered nurse prescriber’ (RNP) for all Finnish nurse prescribers, regardless of educational background.

Finnish nurse prescribing legislation has significantly developed and expanded since May 2019. The context in which nurse prescribing can be practised has expanded from primary to specialised care [25], and the number of included conditions and medications has increased [26]. Part of this reform entailed introducing governmental financial support for nurse prescribing education [27]. The coronavirus [COVID-19] pandemic occurred during the reform. The Finnish healthcare sector adapted rapidly to the pandemic by increasing the use of remote consultations [17], and by temporarily transferring a fourth of the Finnish RN workforce to other tasks or units [28]. These strategies altered the conditions for RNPs, whose prescriptive authority was bound to their workplaces [23]. In addition, remote nurse prescribing was allowed only for urinary tract infections during the COVID-19 pandemic [16].

An extensive official evaluation of the effects and safety of nurse prescribing conducted by the Ministry of Social Affairs and Health in Finland [24] found that patients were well assessed, the working diagnoses and treatments were correct and the prescriptions offered were safe. There was, however, dissatisfaction with some organisational factors related to the role, such as the procedures required to enable nurse prescribing, inadequate referral of patients to RNPs and resistance to role implementation. There were also issues related to salary compensation and the highly restricted prescribing model [24].

There are only three previous research articles on nurse prescribing in Finland [6, 7, 14]. These research articles are all based on data collected before the nurse prescribing reform. In a comparative register study, RNPs’ patients were shown to have fewer readmissions than physicians’ after the initial appointment when a similar antibacterial medication was prescribed [7]. An interview study revealed that the experiences of nurse prescribing in primary outpatient services vary among leaders and health professionals and that opinions regarding the benefits of nurse prescribing are most positive in the student health and family planning contexts [14]. A qualitative study with nurses enrolled in nurse prescribing education showed that awareness of nurse prescribing education among colleagues and a well-defined job description at their workplaces were important facilitators of the development of competence [6]. Since the expansion of nurse prescribing legislation, no study has evaluated the factors influencing the utilisation of nurse prescribing. Thus, it is unclear whether Finnish nurse prescribers have been optimally utilising their prescriptive authority since its introduction 15 years ago [6, 7, 14, 15].

## Methods

### Aim

The aim of this study was to explore factors associated with the utilisation of nurse prescribing in Finland.

### Study design

This was a cross-sectional questionnaire study at the national level that adhered to the Strengthening the Reporting of Observational Studies in Epidemiology guidelines for cross-sectional studies, Additional file 1 [29].

### Study setting

The study was conducted in Finland, a European country with 5.5 million inhabitants. Public healthcare services in Finland are funded by the state [17]. At the time of this study, the public healthcare system was divided into primary and specialised care. The study was carried out at the end of the COVID-19 pandemic in public outpatient healthcare settings—that is, primary and specialised outpatient clinics in hospitals and healthcare centres—and in home healthcare. Outpatient clinics for RNPs constitute independent clinics where they conduct physical examinations, take patient history, order laboratory tests and prescribe medication [19].

### Instrument and its validity and reliability

Given the lack of an instrument that could be directly implemented in the Finnish nurse prescriber context and to achieve the aim of this study, a questionnaire inspired by several instruments in English, Norwegian and Finnish was designed in 2020–2021 [24, 30–33] and adapted to the national context. The questionnaire and information letter were provided in the official languages of Finnish and Swedish. The forward-back translation was inspired by the translation process described in the guidelines for translation in cross-cultural healthcare research [34]. The translation was carried out by the research group, which consisted of experienced researchers representing both national language groups, as well as members with strong Norwegian and English language skills, and was subsequently checked for quality by external professional experts. The research group was familiar with the research area and its terminology. The questionnaire and the information letter were piloted twice with Swedish and Finnish participants. Two experts—an RNP and a developer of Finnish nurse prescribing—participated in the first pilot test, which included an estimation of the time required to complete the questionnaire. Another pilot test was conducted with two RNPs after revisions to the questionnaire, which included adaptation to the COVID-19 pandemic, clarification or supplementation of some concepts, question and answer options, and a future perspective regarding the development of nurse prescribing. Clarifications were provided to improve one question after the second pilot test. According to the recommendations made by the trade union, the information letter was supplemented with instructions for participation during off-duty hours.

In addition to items related to demographic characteristics, the final questionnaire consisted of 26 questions, of which 11 explored the practice patterns of Finnish nurse prescribers according to the competence domains in the Caring Advanced Practice Nursing Model by Fagerström [18]. Six questions covered prescribing patterns, one was related to development needs and eight focused on work satisfaction. For this study, demographic characteristics and questions on practices and prescribing patterns were selected and described in Tables 1-3. Some questions on demographics were inspired by the Ministry of Social Affairs and Health in Finland (educational background, length of prescribing experience, workplace) [24]. Questions on practice patterns were inspired by Henni [31], while prescribing patterns were inspired by Latter et al. (weekly patients prescribed medication) [33]. Questions and answer options were modified from the original instruments. A Finnish doctoral thesis [15] noted several factors that facilitated successful nurse prescribing, some of which were considered in the choice of outcome variables for the present study. A description of the demographic variables included in the study is found in Table 1, while the explanatory variables and outcome variables are presented in Tables 2 and 3, respectively.

**Table 1:** Demographic characteristics of the registered nurse prescribers (*n* = 72)

Demographic characteristics	*n* **(%)**
Gender^a^	
Female	70 (97.2)
Male	2 (2.8)
Age (years)^b^	
25–34	5 (6.9)
35–44	20 (27.8)
45–54	28 (38.9)
55–64	19 (26.4)
Continuing education^c^	28 (38.9)
Employment area where participants were working highest proportion^d^	
General nurse clinic in primary care	35 (52.2)
Emergency department	14 (20.9)
Family planning, sexual and contraceptive clinics	11 (16.4)
Student healthcare	3 (4.5)
Occupational health care	1 (1.5)
Other unit	3 (4.5)
Working time	
Full-time	56 (77.8)
Part-time	16 (22.2)
Typical working days	
Days only	62 (86.1)
All others^e^	10 (13.9)

^a^ Gender: empty category ‘other/do not want to respond’ not included

^b^ Age: empty categories ‘< 25’ and ‘> 64 years’ not included

^c^ Minimum 30 European Credit Transfer and Accumulation System credits in addition to nurse prescribing education

^d^ Working time divided between employment areas. Participants who reported 50/50 excluded (*n* = 5)

^e^ The merged category ‘All others’ consists of days and evenings (*n* = 7); days, evenings, and nights (*n* = 2); and other (*n* = 1)

**Table 2:** The registered nurse prescribers (*n* = 72) explanatory variables used in Table 4 and 5

Explanatory variables	n (%)
Education	
Registered nurse	30 (41.7)
Specialised nurse^a^	30 (41.7)
Master’s degree	12 (16.7)
Work experience as a registered nurse or specialised nurse^a^ (years)	
6–15	31 (43.1)
16–25	25 (34.7)
26–35	16 (22.2)
Work experience of nurse prescribing (years)	
0.1–3.4	34 (47.2)
3.5–6.9	20 (27.8)
7.0–10.3	18 (25.0)
Assessment of pharmaceutical interactions and contraindications on a 1–4 scale^b^	
Never/a few times per year (1)	4 (5.6)
Monthly (2)	11 (15.3)
Weekly (3)	27 (37.5)
Daily (4)	30 (41.7)
Instructions for referral^c^	
Yes	50 (69.4)
No	22 (30.6)

^a^ Registered nurse prescribers with a nursing education longer than 3.5 years: midwives, public health nurses, paramedics or specialist nurses

^b^ The categories *never* and a *few times per year* were merged

^c^ Existing instructions in the organisation for the referral of patients to registered nurse prescribers

**Table 3:** The registered nurse prescribers (*n* = 72) outcome variables used in Table 4 and 5

Outcome variables	
Weekly patients prescribed medication^a^ (*n* (%))	
< 1 (Category 1)	4 (5.6)
1–5 (Category 2) (Median)	33 (45.8)
6–10 (Category 3)	23 (31.9)
11–20 (Category 4)	10 (13.9)
21–40 (Category 5)	2 (2.8)
Length of prescribing consultation (minutes) (*n* (%))	
15 (Category 1)	5 (6.9)
20 (Category 2)	17 (23.6)
30 (Category 3) (Median)	29 (40.3)
45 (Category 4)	13 (18.1)
60 (Category 5)	8 (11.1)
Level of nurse prescribing utilisation^b^ (scale 1–8)^c^	
Mean	5.25
Median	6.00
SD	2.05
Degree of referral^d^ (scale 1–8)^c^	
Mean	5.43
Median	6.00
SD	1.89

^a^ Weekly patients who are prescribed medication

^b^ The level of utilisation of nurse prescribing at the participant’s workplace

^c^ Categories from 1 to 8: 1 = to a small degree; 8 = to a large extent

^d^ The degree of referrals of patients who could benefit from nurse prescribing to the registered nurse prescriber clinics

### Sample and data collection

To enable a cross-national sample from all regions in Finland, the participants were recruited by a trade union for health professionals, which supervises its members’ professional interests, and via snowball sampling. All RNPs within Finland were eligible to join. There were no exclusion criteria. There were 616 RNPs in total in Finland in September 2021 by the end of the data collection (Valvira, the National Supervisory Authority for Welfare and Health in Finland), email, 2021 Sep 10). The data collection focused on total population sampling. However, the exact response rate is unclear because of the recruitment methods. Not all eligible RNPs could be reached through the trade union; therefore, the exact number of invited individuals remains unknown. Based on the total number of submitted questionnaires (*n* = 84) and the total number of Finnish RNPs registered at Valvira by the end of data collection, the response rate would have been at least 13.6%. The power analysis with the 72 participants who were working as RNPs and included in the analysis showed that 1-β was 0.55—that is, only 55% of the significant results were significant with this sample size [35].

The data were collected between June 4 and September 12, 2021 via an online questionnaire created using E-lomake software [36]. The trade union sent the study invitation via e-mail to 589 RNPs’ e-mail addresses in June 2021, and three reminders were sent 2, 10 and 12 weeks later. To increase attention, the study information and reminders were also published in the trade union’s social media and internet forums. The trade union recruitment was supplemented with invitations sent to a social media group for RNPs in August 2021 and to a group of RNPs in a Swedish-speaking region due to the low response rate among Swedish-speaking RNPs. The participants selected via snowball sampling were encouraged to share the study invitation with their RNP colleagues.

### Data analysis

Statistical analysis was conducted using jamovi software versions 2.3.21 and 2.4.8 [37] and SPSS version 28.0 [38]. The categorical variables are presented as frequencies and percentages. Medians are reported for the outcome variables. There were no missing values in the variables included in any of the multiple regression analyses.

The aim of this study—to explore factors associated with the utilisation of Finnish nurse prescribing—was operationalised for six multiple linear regression models with three separate outcome variables: ‘weekly patients prescribed medication’, ‘level of nurse prescribing utilisation’ and ‘degree of referral’. All three outcome variables had the same two sets of four explanatory variables, differing from each other for only one explanatory variable. All explanatory variables were entered into the models as fixed factors, and they are presented as regression coefficients with 95% confidence intervals and their *p*-values in three categories. Results with *p*-values (*p*) ≤ 0.05 were considered statistically significant.

Of the three ordinal scale outcome variables, the ‘level of nurse prescribing utilisation’ and ‘degree of referral’ were Likert-type scales with eight category variables (1 = *to a small degree*, 8 = *to a large extent*) and were treated as interval scale outcome variables. The ‘weekly patients prescribed medication’ was a five-category variable whose frequencies of categories were normally distributed visually and based on skewness and kurtosis. Its category division closely resembled a geometric series; the upper limit roughly doubled when moving from one category to the next. Ordinal regression analysis with logit link function was also conducted for this ordinal scale outcome variable, and the exact same categories of the same three explanatory variables became statistically significant predictors. The test of parallel slopes fulfilled the condition of *p* > 0.05. However, there were several cells with zero frequencies. For the sake of consistency, only the results of the linear regression are presented in more detail. The assumptions of the linear regression analysis (normality, homoscedasticity and independence of residuals; no multicollinearity; and no excessive outliers) were checked and fulfilled.

For an in-depth evaluation of the role of the variable ‘length of prescribing consultations’, an additional multiple linear regression analysis was performed with this variable as an outcome variable and three others as explanatory variables. The outcome variable had cases in five categories (see Table 3), corresponding to values of 1–5. In the Results section, some regression coefficients are reported consistently as points, not as category changes. In each model, the strongest predictor of the outcome variable was determined by the explanatory variable with the highest statistically significant regression coefficients (in absolute values) in its categories.

### Ethical considerations

The ethical guidelines of the Finnish National Board on Research Integrity TENK [39] and the World Medical Association Declaration of Helsinki [40] were followed. Ethical permission was granted by the Board for Research Ethics at Åbo Akademi University on March 9, 2021. Permission to use the included instruments was granted orally or by email. A trade union was chosen for data collection because of its ability to conduct a national study with participants scattered geographically across different organisations. The study was approved by the trade union in April 2021. Due to the recruitment method, research permit applications were not sent to the participants’ employers, and participation during off-duty hours was recommended. The participants were informed that they could give their informed consent by submitting the questionnaire in an information leaflet, which also described data management, voluntariness, secrecy and the right to withdraw from the study. The data will be managed and stored confidentially for 10 years according to the European Union General Data Protection Regulation 2016/679 [41] and the Data Protection Act 1050/2018 [42] and will thereafter be deleted. The raw data, which are stored confidentially and have continuous backup, are available only for researchers who are involved in the current research.

## Results

A total of 84 participants from 16 of the 19 regions in Finland responded to the questionnaire and took part in the study. Of these, 69 (85.7%) worked as RNPs, and three (3.6%) were temporarily transferred to other tasks due to the COVID-19 pandemic. A total of 12 (14.3%) participants were not prescribing. Of these, 7 (8.3%) had prescribed previously, and 5 (6.0%) had never prescribed after completing their nurse prescribing education. The demographic characteristics of the RNPs (*n* = 72) in the final study population are presented in Table 1, while the characteristics of the explanatory and outcome variables are presented in Tables 2 and 3.

Six multiple linear regression models with three outcome variables related to the utilisation of Finnish nurse prescribing are presented in Table 4. The models explained approximately 24% of the variation in the outcome variables. The length of prescribing consultations was found to be associated with all the outcome variables included in Table 4. In most models, time categories other than the reference 15-minute consultations were associated with greater utilisation of nurse prescriptive authority. Regarding the degree of nurse prescribing utilisation (Models 2a–b) and degree of referral (Models 3a–b), 60-minute consultations were found to be the strongest predictor of a higher level of utilisation and a higher degree of referral—both outcome variables increased with an average of about three points on a scale of 1–8. The 30-minute consultations were found to be the strongest predictor of a larger number of weekly patients who were prescribed medication (Models 1a–b). The number of patients who were prescribed medication increased significantly by about one category—that is, roughly doubled. Nurse prescribing experience longer than 3.4 years was significantly associated with an increased number of patients who were prescribed medication and a higher degree of utilisation and referral, except in Model 3a. Length of work experience as an RN or specialist nurse was not associated with any of the outcome variables. Compared to the reference RN education, the RNPs with specialised nursing education reported a half category higher frequency of weekly patients who were prescribed medication (Model 1a)—that is, about one and a half times. Specialised nursing education was also associated with an approximately 1.25-point increase in referral on a scale of 1–8 (Models 3a–3b). The existing instructions for referral to RNPs were found to be associated with a greater degree of referral, with an increase of 1.01 points on a scale of 1–8 (Model 3b).

**Table 4:** Multiple linear regression models of registered nurse prescriber’s (*n* = 72) utilisation of prescriptive authority

Model 1a–3b, Outcome variables	1a. **Weekly patients prescribed medication**^**a**^			2a. **Degree of nurse prescriptive authority utilisation**^**a**^			3a. **Degree of referral**^**a**^			1b. **Weekly patients prescribed medication**^**a**^			2b. **Degree of nurse prescriptive authority utilisation**^**a**^			3b. **Degree of referral**^**a**^		
	**Adj. R**^***2***^** = .258, p=.001**			**Adj. R**^***2***^ ** = .218, p = 0.004**			**Adj. R**^**2**^ ** = .215, p = 0.004**			**Adj. R**^***2***^ ** = .250, p = 0.001**			**Adj. R**^***2***^ ** = .246, p = .001**			**Adj. R**^***2***^ ** = .260, p = < .001**		
**Explanatory variables**	**B**	**95% CI**	*p*	**B**	**95% CI**	*p*	**B**	**95% CI**	*p*	**B**	**95% CI**	*p*	**B**	**95% CI**	*p*	**B**	**95% CI**	*p*
Intercept^b^	1.37	.60–2.1	** < .001**	1.32	−.49–3.12	.150	2.05	.38–3.71	.017	1.32	.54–2.09	.001	.96	−.83–2.75	.287	1.52	−.10–3.15	.066
Education (ref. RN^c^)																		
Specialised nurse^d^	**.51**	**.03–.99**	**.039**	.61	−.52–1.7	.287	**1.25**	**.20–2.29**	**.020**	.46	−.01–.93	.056	.56	−.53–1.64	.308	**1.24**	**.25–2.22**	**.015**
Master’s degree	.25	−.32–.82	.389	.44	−.91–1.79	.520	.66	−.59–1.89	.300	.24	−.34–.83	.404	.55	−.78–1.89	.411	.86	−.36–2.08	.163
Work experience as a RN^c^, or specialised nurse^d^ (years) (ref. 6–15)																		
16–25	.05	−.40–.50	.830	−.08	−1.14–.97	.876	−.67	−1.67–.27	.156	–	–	–	–	–	–	–	–	–
26–35	.39	−.22–.98	.203	.61	−.80–2.02	.391	−.36	−1.66–.94	.584	–	–	–	–	–	–	–	–	–
Work experience of nurse prescribing (years) (ref. 0.1–3.4)																		
3.5–6.9	**.57**	**.08–1.06**	**.024**	**1.19**	**.03–2.34**	**.044**	.90	−.16–1.97	.096	**.68**	**.21–1.15**	**.005**	**1.55**	**.47–2.64**	**.006**	**1.22**	**.23–2.20**	**.017**
7.0–10.3	.04	−.56–.64	.894	**1.46**	**.05–2.86**	**.042**	.89	−.40–2.19	.174	.26	−.25–.77	.309	**1.89**	**.73–3.06**	**.002**	.85	−.21–1.91	.114
Length of prescribing consultations (minutes) (ref. 15)																		
20	.43	−.39–1.25	.296	**2.92**	**1.00–4.85**	**.004**	**3.08**	**1.30–4.86**	** < .001**	.40	−.46–1.25	.357	**2.47**	**.51–4.4**	**.014**	**2.34**	**.56–4.13**	**.011**
30	**1.07**	**.29–1.86**	**.008**	**3.00**	**1.15–4.86**	**.002**	**2.55**	**.85–4.26**	**.004**	**1.12**	**.32–1.93**	**.007**	**2.76**	**.90–4.61**	**.004**	**1.92**	**.23–3.60**	**.027**
45	.65	−.25–1.56	.155	**2.96**	**.82–5.10**	**.007**	**2.71**	**.73–4.68**	**.008**	.75	−.17–1.67	.110	**2.78**	**.66–4.91**	**.011**	**2.14**	**.20–4.07**	**.031**
60	.73	−.25–1.72	.142	**3.35**	**1.03–5.68**	**.005**	**3.27**	**1.13–5.41**	**.003**	.82	−.18–1.83	.107	**3.10**	**.78–5.42**	**.010**	**2.58**	**.46–4.69**	**.018**
Instructions for referral^e^ (ref. no.)	–	–	–	–	–	–	–	–	–									
Yes	–	–	–	–	–	–	–	–	–	.07	−.36–.50	.744	.75	−.24–1.75	.136	**1.01**	**.098–1.92**	**.030**

*Note.* Abbreviations: Adj.*R*^*2*^ = Adjusted *R*^*2*^ in multiple linear regression model; 95% CI = 95% confidence interval of regression coefficient; B = regression coefficient. Regression coefficients and 95% CI with statistically significant *p*-values (*p* ≤ 0.05) in bold

^a^ See Table 3 for outcome variable characteristics and description of classes and Likert scales

^b^ Represents the reference level

^c^ RN = registered nurse

^d^ Registered nurse prescribers with a nursing education longer than 3.5 years: midwives, public health nurses, paramedics or a specialist nurse

^e^ Existing instructions in the organisation for referral of patients to the registered nurse prescribers

The length of prescribing consultations was further analysed as an outcome variable using multiple linear regression (Table 5). All explanatory variables were found to be significantly associated with the length of prescribing consultations (*p* < 0.05). Specialised nursing education or a master’s degree was associated with longer consultations—almost one point higher—corresponding to roughly 40% longer consultations than those by RNPs with an RN education. Nurse prescriber work experience longer than 3.4 years was associated with shorter consultations. A more frequent assessment of pharmaceutical interactions and contraindications was associated with longer consultations, and daily frequency was the strongest predictor in the model, with a large effect on the length of consultations and an increase of 1.86 points, corresponding to roughly 80% longer time spent in consultation.

**Table 5:** Multiple linear regression model of the registered nurse prescriber’s (*n* = 72) length of prescribing consultations

Outcome variable	**Length of prescribing consultations**^**a**^		
	Adj. R2 = .335, p = < .001		
**Explanatory variables**	**B**	**95% CI**	*p*
Intercept^b^	**1.50**	**.58–2.42**	**.002**
Education (ref. RN^c)^			
Specialised nurse^d^	**.80**	**.29–1.30**	**.002**
Master’s degree	**.97**	**.35–1.60**	**.003**
Work experience of nurse prescribing (years) (ref. 0.1–3.4)			
3.5–6.9	**−.77**	**−1.28–−.27**	**.003**
7.0–10.3	**−.56**	**−1.11–−.01**	**.046**
Assessment of pharmaceutical interactions and contraindications (ref. never/a few times per year)			
Monthly	**1.33**	**.29–2.36**	**.013**
Weekly	**1.09**	**.14–2.04**	**.025**
Daily	**1.86**	**.90–2.82**	** < .001**

*Note.* Abbreviations: Adj.*R*^*2*^ = Adjusted R^2^ in multiple linear regression model; 95% CI = 95% confidence interval of regression coefficient; B = regression coefficient

Regression coefficients and 95% CI with statistically significant *p*-values (*p* ≤ 0.05) in bold

^a^ Length of prescribing consultation is expressed in classes 1–5. See Table 3 for outcome variable characteristics

^b^Represents reference level

^c^RN = registered nurse

^d^Registered nurse prescribers with a nursing education longer than 3.5 years: midwives, public health nurses, paramedics or a specialist nurse

## Discussion

This study found that longer prescribing consultations were associated with a higher degree of utilisation of nurse prescribing. However, the RNP consultations shortened with longer work experience, and their weekly number of patients who were prescribed medications increased. A longer background education than the registered nurse education increased the accuracy of correctly referred patients to RNPs clinics and the frequency of patients who were prescribed medication.

This study also found that RNPs reported the degree of nurse prescribing utilisation as moderately high after the reform. Similarly, a recent Finnish report found that RNPs use their advanced knowledge in every patient encounter [43]. Nonetheless, few of their weekly patients received medication prescriptions (median: 1–5 patients/week), suggesting that their nurse prescriptive authority was not utilised to its full potential. The results aligns with a report in which register data showed that Finnish RNPs, on average prescribed 34 reimbursable medications (excluding contraceptives and over the counter medications) in the year of the data collection [43]. The low volume of patients who were prescribed medication in this study also aligns with results from Sweden and Spain, which apply limited nurse prescribing models [8, 9] that are less comprehensive than the Finnish model [5]. One reason for the low volume of patients who were prescribed medication may be that the limited prescribing model is structured with too few included medications, which previous research has identified as a factor that negatively affects nurse prescribers´ ability to utilise their advanced competence to its full potential [13]. Furthermore, dependence on the medical profession can be barrier to implementing nurse prescribing [6, 13]. The Finnish nurse prescribing model is limited in several ways, and these limitations negatively affect RNPs’ ability to prescribe [15, 43, 44].

We found that a specialised nursing background was associated with a higher number of weekly patients who were prescribed medication [8, 10] compared to an RN background. Our findings show that RNPs with specialised nursing education reported higher accuracy of patient referrals to their clinics than RNPs with other educational backgrounds. Similarly, a previous study showed that the majority of contraceptives are prescribed by Finnish RNPs in student healthcare services and family planning clinics, where specialised nurses often work [15]. According to that study, the RNPs in these units have clearly defined roles that differ from other nursing roles; this facilitates the appropriate referral of patients. However, our results indicate that the degree of nurse prescribing utilisation was not explained by specialist nursing or master’s degree education, which can be interpreted as the organisation using the RNP’s specialised nurse competence (their educational background) instead of the nurse prescriptive authority. This finding implies the need for a greater awareness of the role of RNPs in organisations, which has also been reported in other studies [6, 43].

Longer prescribing consultations were associated with factors related to a higher level of utilisation of nurse prescribing. The number of patients prescribed medication doubled when consultation durations increased from 20 to 30 minutes. In this study, the median length of consultations was 30 minutes, but longer consultations were associated with a higher number of weekly patients who were prescribed medication, a higher degree of utilisation of nurse prescribing and improved referral to RNPs. These results suggest that sufficient time is needed for prescribing and that 15-minute consultations are too short. There are no studies focusing on consultation lengths among nurse prescribers in countries that apply a limited prescribing model, but an international systematic review pointed out that additional time must be reserved for prescription-related decision-making [3].

However, longer consultations can decrease economic benefits and reduce the number of consultations as the theoretical cost-effectiveness of Finnish RNPs depends on their working time in direct clinical patient care with patients who benefit from nurse prescribing and on the length of their prescribing consultations [45]. Our findings suggest that instructions for the referral of patients to RNPs clinics significantly influence the degree of referral but not the degree of nurse prescribing utilisation or the number of weekly patients who are prescribed medication. This aligns with a previous finding that for patients to be referred correctly, instructions for their referral to RNPs are required [15]. However, our results contradict a report that found that leaders of healthcare organisations believe that inadequate instructions for the referral of patients are related to the low utilisation of nurse prescribing [44].

An explanatory factor for longer prescribing consultations was educational background as a specialised nurse or a master’s degree. The results should be further analysed to determine whether these RNPs patient groups and practice patterns differ from those of RNs. It is possible that more educated nurses receive referrals of patients who have more complex medical backgrounds and care needs to their clinics, that require additional time. The daily assessment of pharmaceutical interactions and contraindications was another explanatory factor for longer prescribing consultations. The evaluation of medication is needed for safe and competent prescribing [1], which requires more time, according to our study.

An important finding of this study was that longer work experience in nurse prescribing was crucial for several outcome variables related to greater utilisation of nurse prescribing: a higher frequency of weekly patients who are prescribed medication, shorter prescribing consultations, a higher degree of nurse prescribing utilisation and the referral of patients. This study confirms an association between longer work experience and shorter prescribing consultations. Longer prescribing experience has previously been indicated as a potential factor that could shorten consultation length and reduces costs [45]. At the beginning of their careers, RNPs should be allowed more time for consultations, with the knowledge that these consultations become shorter with increased experience. This study further revealed that measures are needed to increase the referral of correct patients to early-career RNPs to facilitate a higher level of nurse prescriptive authority utilisation, which could also increase the probability that they will remain in the profession. Beyond the availability of instructions for referral to RNPs revealed in our study, consultation support, mentorship and continuing education should be considered in the development of support for early-career RNPs [8]. The consultation support that physicians are obliged to offer RNPs is often insufficient, making prescribing slower and negatively affecting the utilisation of nurse prescribing, which motivates further development of support for RNPs [44]. One solution could be to continue trainee supervision with a physician or more experienced RNP as a mentorship strategy after receiving the nurse prescriptive authority.

In February 2023, there were 774 qualified nurse prescribers in Finland, of which 691 were registered as RNPs. This corresponds to approximately 1% of the Finnish RN nurse workforce [20]. The government financially supports nurse prescribing education [27]. Nevertheless, this study shows that 14% of qualified RNPs were not working as RNPs, and 6% never started their RNP careers. The reasons for why these nurses did not work in careers corresponding to their education were further explored in another unpublished manuscript of the authors [46]. More qualified RNPs utilised their prescriptive authority in this study than in a later report [44], in which as few as 66% of Finnish RNPs utilised their prescriptive authority. Finnish RNPs are experienced and have short working careers owing to retirement or career progression into administrative roles [19], which can be a reason why they do not utilise their prescriptive authority. Furthermore, a lack of personnel resources has been shown to be a barrier to RNPs working in roles that correspond to their competence [44].

Since 2021, there have been minor changes in the legislation regulating nurse prescribing. RNPs’ prescriptive authority has been extended to outpatient clinics in the wellbeing services counties in which they are working [23] due to a national health care reform [17]. RNPs are allowed to change the dosage instruction of a medication according to a written order and instructions of a physician [47]. There is a proposal in the Parliament of Finland to allow nurse prescribing via telephone and remote consultations beginning in 2026, while the limited prescribing model still remains unchanged [48]. To optimise the utilisation of Finnish nurse prescribing, it is crucial to increase awareness of the role of RNPs in organisations and clearly define instructions for the referral of patients to RNPs, particularly those with an RN education or a master’s degree. Furthermore, it is important to reduce some of the limitations of nurse prescriptive authority and to give RNPs greater autonomy so that more patients can benefit from the service. There is also a need to support early-career RNPs in their prescribing roles. A mentorship programme could strengthen these nurses’ ability to utilise their prescriptive authority more.

There is a need for additional research on nurse prescribing in European countries that apply a limited nurse prescribing model, as there is a lack of internationally published research from several of these countries. Further research on nurse prescribing in the Finnish context is also needed. The findings of the present study indicate the need for additional knowledge of how to support RNPs in the Finnish healthcare system to facilitate the utilisation of their prescriptive authority. In addition, more knowledge about how to support early-career RNPs is needed. The length and content of RNPs’ consultations in terms of clinical activities, practice patterns and patient groups should be further studied. In addition, a comparison between RNPs’ and physicians’ consultations in terms of length, content and patient results should be conducted.

### Strengths and limitations

This was the first Finnish nurse prescriber study with a national perspective that aimed to explore the factors associated with the utilisation of nurse prescribing. This study was conducted during the COVID-19 pandemic, which may have affected the number of responses, the results and the number of patients who were prescribed medication each week. A reduction in physical consultations [17] could have reduced RNPs’ ability to prescribe, while transfers to other workplaces or to tasks outside outpatient settings [28] could have restricted their legal ability to utilise their prescriptive authority. Three RNPs included in the final study population were temporarily transferred to other tasks and answered the questions as the situation was before the pandemic. The low sample size and the unknown exact response rate are limitations of this study. The explanatory power of the study was moderately good.

Despite the unknown response rate, the results of the study are consistent with or comparable to those of other studies conducted in European countries that apply a limited prescribing model. The responses were self-reported, and some outcome variables may have differed from the actual levels. All models included in Table 4 had uniform results. Both ordinal and linear regression analyses of the outcome variable ‘weekly patients prescribed medication’ yielded identical results of statistical significance. Nevertheless, the results of models 1a and 1b should only be considered indicative because the outcome variable ‘weekly patients prescribed medication’ had only five categories, while the other two variables had scales of 1–8.

Although we included two pilot tests, further reliability and validity testing of the questionnaire are needed. To reduce the risk of bias, our data were collected by a trade union. The data were supplemented with snowball sampling, which could have increased the number of responses in a given region.

## Conclusion

The current study focused on factors related to the utilisation of nurse prescribing in Finland after the national nurse prescribing reform. The findings suggest that RNPs’ prescriptive authority is not being fully utilised a decade after its introduction and that measures are needed to raise awareness of the role of RNPs in the Finnish healthcare system. The number of patients who were prescribed medication by RNPs was quite low, even though the RNPs experienced that their prescriptive authority was utilised to a moderately high degree. This may have been a consequence of the Finnish hybrid strategy during the COVID-19 pandemic, combined with the limits of the Finnish nurse prescriptive authority. RNPs were able to utilise their competence, but prescribing was restricted. Instructions for the referral of patient groups to RNPs can increase the number of patients correctly referred to RNPs. However, these instructions do not affect the degree of nurse prescribing utilisation or the number of weekly patients prescribed medication. The limitations of the prescribing model were assessed as a factor that reduces the number of patients who could benefit from the services.

The utilisation of nurse prescribing increases with longer prescribing experience. For RNPs to remain in the profession and utilise their prescriptive authority to a greater extent, they need to be supported early in their careers. Moreover, prescribing requires sufficient time, especially during the first years of the RNP career, which should be considered when scheduling outpatient appointments. The results contribute to the understanding of nurse prescribing utilisation in countries that apply limited prescribing models and provide insights for developing nurse prescriber services in Finnish, European and global contexts.

## Data Availability

Raw data are not publicly accessible to protect the integrity of the participants. The survey data are confidentially managed and stored according to the European Union General Data Protection Regulation 2016/679 and the Data Protection act 1050/2018.

## Abbreviations

COVID-19: Coronavirus disease 2019
ECTS: European Credit Transfer and Accumulation System
RN: Registered nurse
RNP: Registered nurse prescriber
UAS: Universities of applied sciences

## Declarations

### Permission to use instruments

Permission to use the included instruments in the questionnaire was given orally or by e-mail.
